# Baicalein prevents stress-induced anxiety behaviors in zebrafish model

**DOI:** 10.3389/fphar.2022.990799

**Published:** 2022-10-31

**Authors:** Logesh Kumar Selvaraj, Srikanth Jeyabalan, Ling Shing Wong, Mahendran Sekar, B. Logeshwari, S. Umamaheswari, Sree Premkumar, Roshan Tej Sekar, M. Yasmin Begum, Siew Hua Gan, Nur Najihah Izzati Mat Rani, Kumarappan Chidambaram, Vetriselvan Subramaniyan, Adel Al Fatease, Ali Alamri, Kathiresan V. Sathasivam, Siddharthan Selvaraj, Kamini Vijeepallam, Shivkanya Fuloria, Neeraj Kumar Fuloria

**Affiliations:** ^1^ Department of Pharmacology, Sri Ramachandra Faculty of Pharmacy, Sri Ramachandra Institute of Higher Education and Research (DU), Chennai, Tamil Nadu, India; ^2^ Faculty of Health and Life Sciences, INTI International University, Nilai, Malaysia; ^3^ Department of Pharmaceutical Chemistry, Faculty of Pharmacy and Health Sciences, Royal College of Medicine Perak, Universiti Kuala Lumpur, Ipoh, Perak, Malaysia; ^4^ Department of Pharmaceutics, College of Pharmacy, King Khalid University, Abha, Saudi Arabia; ^5^ School of Pharmacy, Monash University Malaysia, Subang Jaya, Selangor, Malaysia; ^6^ Faculty of Pharmacy and Health Sciences, Royal College of Medicine Perak, Universiti Kuala Lumpur, Ipoh, Perak, Malaysia; ^7^ Department of Pharmacology, College of Pharmacy, King Khalid University, Abha, Saudi Arabia; ^8^ Faculty of Medicine, Bioscience, and Nursing, MAHSA University, Jenjarom, Selangor, Malaysia; ^9^ Faculty of Applied Sciences, AIMST University, Bedong, Kedah, Malaysia; ^10^ Faculty of Dentistry, AIMST University, Bedong, Kedah, Malaysia; ^11^ Faculty of Pharmacy, AIMST University, Bedong, Kedah, Malaysia; ^12^ Center for Transdisciplinary Research, Department of Pharmacology, Saveetha Institute of Medical andTechnical Sciences, Saveetha Dental College and Hospital, Saveetha University, Chennai, Tamil Nadu, India

**Keywords:** baicalein, zebrafish, anti-anxiety, anti-stress, neuroprotection

## Abstract

Baicalein is a flavonoid mainly obtained from plants with wide range of biological activities, including neuroprotection. An acute and unexpected chronic stress (UCS) protocol has recently been adapted to zebrafish, a popular vertebrate model in brain research. The present study was aimed to evaluate baicalein’s anti-anxiety potential in a zebrafish model by induction, which included neuropharmacological evaluation to determine behavioural parameters in the novel tank diving test (NTDT) and light-dark preference test (LDPT). The toxicity was also assessed using the brine shrimp lethality assay, and the 50% lethal concentration (LC_50_) was determined. The animals were then stressed for 7 days before being treated with different doses of baicalein (1 and 2 mg/L) for another 7 days in UCS condition. Due to acute stress and UCS, the frequency of entries and time spent in the 1) top region and 2) light area of the novel tank reduced significantly, indicating the existence of elevated anxiety levels. The biological activity of baicalein was demonstrated by its high LC_50_ values (1,000 μg/ml). Additionally, baicalein administration increased the frequency of entries and duration spent in the light region, indicating a significant decrease in anxiety levels. Overall, the present results showed that baicalein has a therapeutic advantage in reversing the detrimental consequences of UCS and acute stress, making it is a promising lead molecule for new drug design, development, and therapy for stress.

## Graphical Abstract

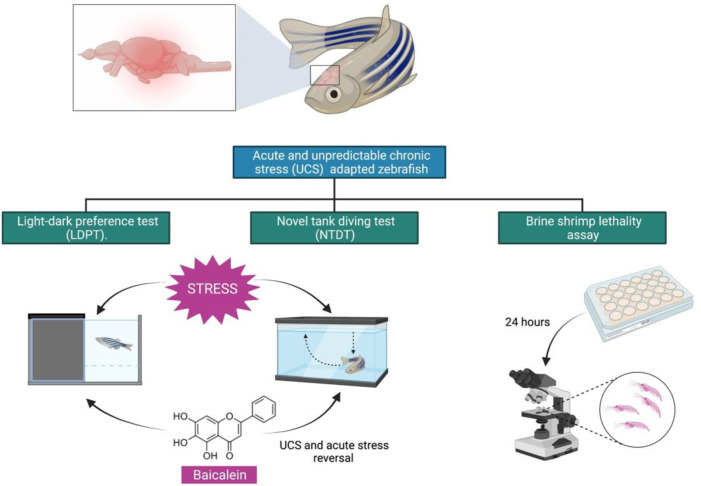

## 1 Introduction

Anxiety is a relatively prevalent behavioural condition in humans and is associated with a traumatic experience (Bystritsky et al., 2013). Stress is a component that can contribute to the development of anxiety disorders and other psychiatric illnesses. Stress is a complex concept to define, though the mechanisms are highly conserved among vertebrates. Therefore, the use of animal models to depict brain problems caused by traumatic events is a valuable tool for developing novel treatments and discovering new drugs (Steimer, 2011).

Alarm pheromone or predator exposure, handling, crowding, social isolation, air exposure, changing water parameters (e.g., pH, salinity and temperature), or bright light exposure can all create acute stress in zebrafish (de Abreu et al., 2021). Additionally, zebrafish may exhibit fear/anxiety-like behaviours in response to acute stressors, such as erratic locomotion (e.g., increased distance and average speed in the tank), freezing, avoidance of light/bright areas and memory deficits (e.g., reduced cognitive performance following alarm pheromone or the presence of a natural predator like the Indian leaf-fish Nandus nandus, exposure) (Stewart et al., 2014a).

**FIGURE 1:**
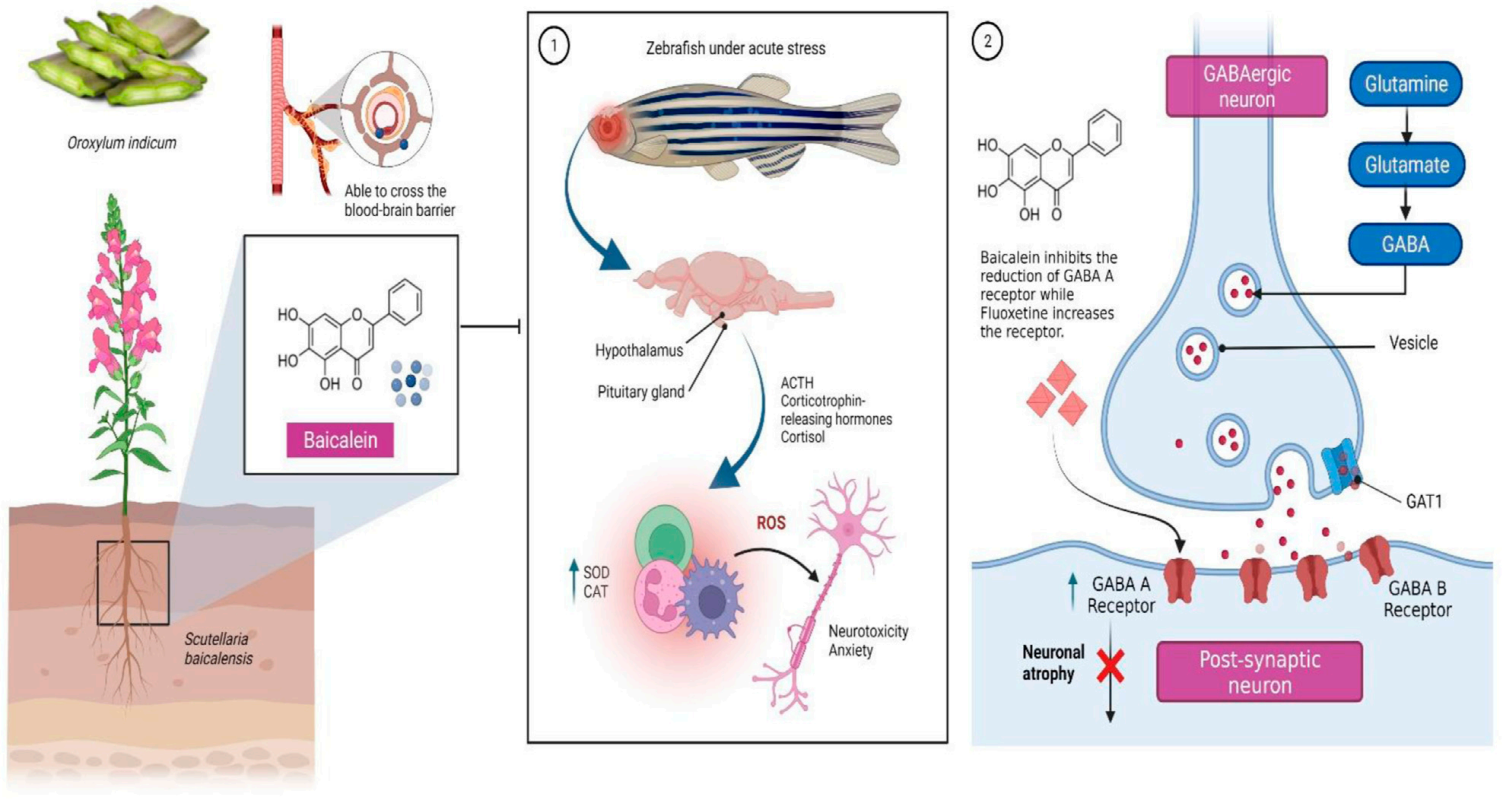
Baicalein, a bioactive molecule found in *Scutellaria baicalensis* Georgi and *Oroxylum indicum* (L.) Kurz, has been shown to be effective in the treatment of neuropsychiatric diseases, particularly anxiety occuring as a result of 1) dysregulation of hormones released from the hypothalamic-pituitary-adrenal (HPA) axis, which when aggravated by a stressor result in chronic oxidative stress and neurotoxicity. It has been suggested that the selective serotonin reuptake inhibitor (SSRI) fluoxetine, which helps to upregulate GABA receptors, may work through a similar mechanism to baicalein to prevent seizures. 2) Baicalein reverses the decline in the expression of the receptors hence may prevent neuronal atrophy. Abbreviations: ACTH, Adrenocorticotropin; SOD, Superoxide dismutase; CAT, Catalase; ROS, Reactive oxygen species; GAT1, GABA transporter 1.

Chronic stress is a significant trigger for the onset of neuropsychiatric diseases and has an increasing relevance in the 21st century (Miller and Raison, 2016). Dysregulation of the hypothalamic-pituitary-adrenal (HPA) axis (Figure 1) and neurotransmitter systems [glutamatergic, noradrenergic, dopaminergic, serotoninergic and gamma-aminobutyric acid or GABA-ergic), decrease in glutathione (GSH) levels, imbalance in oxidative status parameters, activation of neuroinflammatory and apoptosis pathways as well as behavioural changes in response to adverse situations are all part of its neurobiology (Chrousos, 2009; Duman et al., 2016; Niedzielska et al., 2016; Mocelin et al., 2019).

The zebrafish (*Danio rerio*) is a model organism frequently used to investigate behavioural and neurochemical aspects of stress-related neuropsychiatric diseases. When compared to mammalian counterparts, this species demonstrates a high degree of genetic and physiological conservation, with several brain regions showing comparable activities (Kalueff et al., 2014). Memory processing is controlled by the lateral pallium of the telencephalon, while anxiety/fear reactions are controlled by the dorsal habenula (Cheng et al., 2014). The hippocampus and amygdala occupy similar regions in the brain. Furthermore, the zebrafish model is desirable since it expresses all of the major neurotransmitter systems seen in mammals (e.g., dopaminergic, serotonergic, cholinergic and noradrenergic) (Horzmann and Freeman, 2016). Zebrafish have extensive cognitive processing and decision-making methods and they are extremely sensitive to pharmacological drugs that modify behavioural functions (Khan et al., 2017). When exposed to stressors that are pharmacologically responsive to anti-stress medications, the species exhibits strong adverse reactions (e.g., anxiety/fear-like behaviours) (Lezak et al., 2017). Stress hormone levels and oxidative stress-related indicators might be useful tools to compare with behavioural data when examining anxiety/fear responses resulting from aberrant stress-related physiology. Several studies have shown that zebrafish models are increasingly useful in the investigation of behavioural, neurochemical, physiological and epigenetic impacts of stress (Nathana et al., 2019). Thus, the zebrafish is an excellent animal model for determining the genetic roots for human stress physiology. When animals respond to a challenge, stress occurs as a response to threat, challenge, or physical and psychological barrier, whereas fear and anxiety are basic emotions that help to ensure safety (Ulrich-Lai et al., 2016).

As mentioned, fear is a cognitive response to an impending threat in the clinical literature, whereas anxiety is an emotional response to fear (Peter et al., 2000). Thus, the inability to suppress fear reactions is a major contributor to both anxiety and stress disorders (Radulovic and Lynn, 2019). The conceptions of fear and anxiety are more precisely defined in the neuroscience literature. Anxiety can be elicited by several potentially dangerous events, whereas fear is elicited by a genuine threat. In zebrafish, certain stresses cause higher levels of anxiety and fear-like responses. Both acute conspecific alarm substance (CAS) exposure and net chasing for example, can elicit fleeing and unpleasant behaviours (Mocelin et al., 2015). Furthermore, CAS promotes protracted defensive behaviours and elevates c-fos expression in the habenula, resulting in a persistent fear-like response (Caio et al., 2018). Since chemical and mechanical stressors are very dissimilar, pharmaceutical therapies aimed at preventing certain stress-induced phenotypes become important.

Baicalein (5,6,7,-trihydroxyflavone), one of the most active flavonoid from natural product, is found in the dried roots of *Scutellaria baicalensis* Georgi (Family: Lamiaceae) and *Oroxylum indicum* (L.) Kurz (Family: Bignoniaceae)*.* To date, several researches have investigated the anti-inflammatory, antioxidant, anti-proliferative, anti-apoptotic and anti-tumor characteristics of baicalein. Baicalein has been reported to pass the blood-brain barrier, thereby having direct pharmacological effects in the brain nuclei (Zhou et al., 2015), making natural products important viable source of novel anxiolytics. For this purpose, identification of phyto constituents become important. For this purpose, rodent models can be used, though expensive. Furthermore, tests using rats are expensive and require a large number of samples (Muniandy, 2018). As a result, the development and utilisation of various animal models are beneficial. Zebrafish have a number of advantages especially in screening natural products. The key advantage is that they can be mass-produced at a low cost. In fact, zebrafish has been confirmed in several studies to be an excellent model for investigating drug molecules with anxiolytic effects (Stewart et al., 2014b). Hence, in this study, the effects of baicalein in preventing fear/anxiety-like behavioural, neurochemical and physiological responses in zebrafish subjected to acute and unpredictable chronic stress are investigated *via* several mechanisms of action.

## 2 Materials and methods

### 2.1 Chemicals

Baicalein as well as other chemicals of analytical grades were purchased from I.L.E Co., Chennai, Tamilnadu, India. Adult wild-type zebrafish were purchased from a local aquarium shop in Kolathur, Chennai, Tamilnadu, India. Authentication of species was done by Dr. D. Sivaraman (Scientist C, Centre for Laboratory Animal Technology and Research, Sathyabama Institute of Science and Technology, Jeppiaar Nagar, Chennai, Tamil Nadu, India).

### 2.2 Animals

Short-fin wild-type (WT) zebrafish (*n* = 200) of equal number of sexes were used. The fish were housed at a maximum density of two fish per litre of water and were acclimatised for 2 weeks prior to the experiment. The fish were fed three times a day with brine shrimp (*Artemia salina*) and received commercial flake fish food on a 14–10-h day/night cycle (lights on at 7:00 a.m.).

### 2.3 Brine shrimp lethality assay

For a wide range of different compounds, a previous study found a significant association between the LC_50_ values for zebrafish embryos and the LD_50_ values for rodents (Ali et al., 2011). Another study that examined toxicity and teratogenicity of a set of compounds and came to a conclusion that zebrafish toxic responses are similar to those of mice (Parng et al., 2002). As a reason, zebrafish embryo toxicity testing looks promising as a preliminary screening technique and perhaps even as a stand-in for mammalian toxicity testing. Baicalein and its derivatives have been carried out for a toxicity study in zebrafish model (Jiang et al., 2018; Zhang et al., 2020; Brinza et al., 2021). No toxicity was observed at the chosen dose/concentration levels, which were selected based on previous investigations that were reported in the literature (Jiang et al., 2018; Zhang et al., 2020; Brinza et al., 2021). Further, to explore toxicity testing, the brine shrimp lethality assay has also been recommended (Rajabi et al., 2015).

*Artemia salina* (150 mg) cysts were incubated for hatching in a conical container (separating funnel) filled with sea water. After 24 h of larvae feeding, yeast solution (0.06%) was added to the hatching chamber which was filled with seawater that was under a constant aeration for 48 h. Subsequently, active nauplii free from egg shells were collected from the chamber and were used for the assay. The cyst was activated after 48 h and the testing will commence when the nauplii reach the II-III larval stages.

From the hatching chamber, 10–15 nauplii were drawn using a Pasteur pipette and were introduced into the 24 well plates. The procedure also necessitates the use of a Pasteur pipette and a microscope. During the larval passage, a volume of no more than 1 ml should be transmitted to avoid affecting the overall volume of the test system (Nachammai et al., 2021). Different concentrations (0.1, 1.0, 10.0, 100.0, and 1,000.0 μg/ml) of baicalein and the positive control (potassium dichromate) were prepared. Subsequently, 0.5 ml was added to each well containing sea water. The plates were maintained at room temperature for 24 h, to allow contact with the active nauplii in the well plates.

The number of surviving nauplii in each well was counted after 24 h. The percentage death was calculated by comparing the mean surviving larvae of the test and control systems. Concentration *versus* percentage lethality is plotted to obtain the 50% lethal concentration (LC_50_) values. The criterion of toxicity is taken as below: LC_50_ values >1,000 μg/ml (non-toxic), ≥500 ≤ 1,000 μg/ml (weak toxicity) and <500 μg/ml (toxic) (Déciga-Campos et al., 2007).

$$Percentage\;of\;death=\left(\frac{Number\;of\;dead\;nauplii}{Number\;of\;dead\;nauplii+Number\;of\;live\;nauplii}\right)X\;100$$

### 2.4 Experimental design and procedures

#### 2.4.1 Unpredictable chronic stress

**FIGURE 2:**
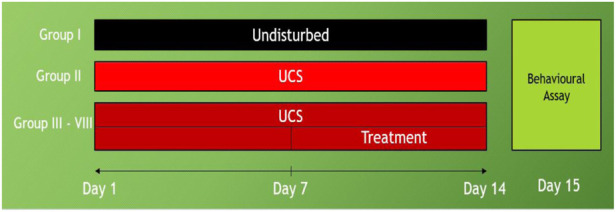
Study timeline of unpredictable chronic stress: Group I- control (non-stressed), Group II -UCS (stressed), Group III- UCS + Diazepam (US1), Group IV- UCS + Group V- Fluoxetine (US2), Group VI- UCS + Group VII- Baicalein conc1 (UB1), Group VIII- UCS + Baicalein conc2 (UB2).

The fish were initially divided into two groups: control (non-stressed), UCS (stressed), UCS + Diazepam (US1), UCS + Fluoxetine (US2), UCS + Baicalein (1 mg/L) (UB1), UCS + Baicalein (2 mg/L) (UB2). The animals were gently changed from their housing tanks to 5L tanks with fresh water (stressed group -UCS) or drug (US1, US2, UB1, UB2) for 10 min daily at 08:00 a.m., culminating in 14 days of UCS and 7 days of treatment (Figure 2).

Stressors were introduced twice daily for a total of 14 days to avoid habituation. The stresses were as follows: using a net to pursue (8 min) –S1, housing tanks had low water levels until the dorsal body wall was visible (2 min) -S2, filling a 250 ml beaker to capacity (50 min) -S3, lowering the temperature of the cooling tank water to 23°C (30 min) -S4, heating tank water to 33°C (30 min) -S5, changing the tank three times in a row with a 30 min interval-S6. Stressors were administered between 8:00 a.m. and 5:00 p.m. The control group was left undisturbed for the duration of the trial, which lasted 14 days. To prevent visual contact of fish from different tanks in the same horizontal plane, a white frosted cardboard (30 × 60 cm) was placed between the tanks (Piato et al., 2011; Marcon et al., 2016).

#### 2.4.2 Acute stress

Animals were divided into different groups (10 animals per group) such as Control (non-stressed group), AS (stressed group), AS + Diazepam (AS1), AS + Fluoxetine (AS2), AS + Baicalein (1 mg/L) (AB1), AS + Baicalein (2 mg/L) (AB2). The control group was transferred to 5L tanks containing fresh water. Immediately after, the animals were subjected to behavioral analyses (NTDT and light/dark test). A video was recorded and was later analyzed by using the ANY-Maze software. Other groups were transferred to 5L tanks containing fresh water. After the 10 min treatment, the fish were chased for 2 min with a net before being subjected to behavioral analyses. The same experimenter executed the net chasing stress in all tests (circular clock-wise movements with the net in the tank, at a regular speed of approximately 40 turns per min) (Aponte and Petrunich-Rutherford, 2019) to ensure consistency.

### 2.5 Behavioral analyses

#### 2.5.1 Novel tank diving test

The animals were individually moved to the novel tank test (NTT) and a video was filmed for 6 min. The ANY-Maze™ software was then used to analyse the videos. A 2.7L tank (24 × 8 × 20 cm) was filled to a height of 15 cm for the innovative tank test. The apparatus was separated into three horizontal zones that were nearly equal in size (bottom, middle and upper). The total distance travelled, the number of crossings between the different zones, the maximum swimming speed, the time spent 1) at the bottom, middle and upper zones of the tank were all analysed for 6 min. In zebrafish, the vertical location in a novel habitat is regarded an anxiety parameter, similar to the thigmotaxic behaviour that rodents tend to exhibit in an open field (Levin et al., 2007).

#### 2.5.2 Light/dark test

A glass tank (18 × 9 × 7 cm) was splitted into two equal sized (dark and white compartments) by using a sliding guillotine-type divider (9 × 7 cm). To allow the zebrafish to swim freely between the two sides of the tank, the water level was raised 3 cm above the tank floor and the divider was lifted 1 cm above the tank floor. The duration spent in the light compartment, the latency to enter the dark compartment and the number of crossings between compartments were all recorded for 5 min after the fish were individually placed in the light zone of the apparatus. The ANY-Maze™ software was then used to evaluate the videos (Ibrahim et al., 2014).

### 2.6 Statistical analysis

The normal distribution of the data was confirmed by D’Agostino-Person tests. The results were analyzed by a One-way analysis of variance (ANOVA) followed by Bonferroni test for multiple comparisons using Graph Pad Prism (Version 8.4.2). The data was expressed as mean ± standard error of mean (S.E.M.). The significance level was set at *p* < 0.05 (Egan et al., 2009).

### 2.7 *In silico* docking analysis

Molecular docking is the estimation of the most effective orientation of the ligand when attached to the receptor. The molecular docking between receptor binding sites and ligands was conducted using the Glide Module of Maestro 12.5 (Schrodinger 2020–3 package). The lowest binding pose of each ligand was maintained. Glide docking scores were performed in three modes 1) High-throughput Simulated Screening (HTVS), 2) Standard Precision (SP) and Extra Precision (XP). The XP mode was used for docking (Shah et al., 2020; Sinha et al., 2020).

The proteins for the docking studies were obtained in pdb format from the Protein Data Bank. For the docking studies, baicalein was obtained from the pubChem chemical database and was stored in a mol format. The target protein disease’s 3D structure was obtained from the RCSB Protein Data Bank. The X-ray crystal co-ordinates for GABA(A) (PDB ID: 1B41) and serotonin transporter (SERT) (PDB ID: 5I73) were obtained from the Protein Data Bank. The standard drugs donepezil and rivastigmine were also acquired and saved in. mol format from the Drug Bank database. For GABA(A) study, diazepam (PubChem ID: 3016) and alprazolam (PubChem ID: 2118) were used as standards, while for SERT study, citalopram (PubChem ID: 2771) and fluoxetine (PubChem ID: 3386) were used as standards. The ligands, including baicalein and standard drugs, were imported into the workspace and were prepared for docking. Baicalein docking scores and patterns were then compared to those seen with standard drugs.

## 3 Results

### 3.1 Brine shrimp lethality assay

**FIGURE 3:**
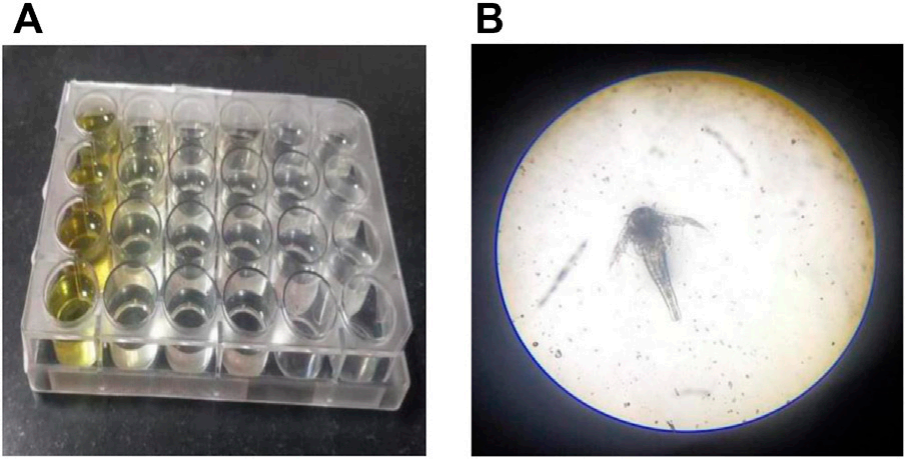
Brine shrimp lethality at 24 h in a 24-well plate. Plate with **(A)** sample and **(B)** brine shrimp.

**FIGURE 4:**
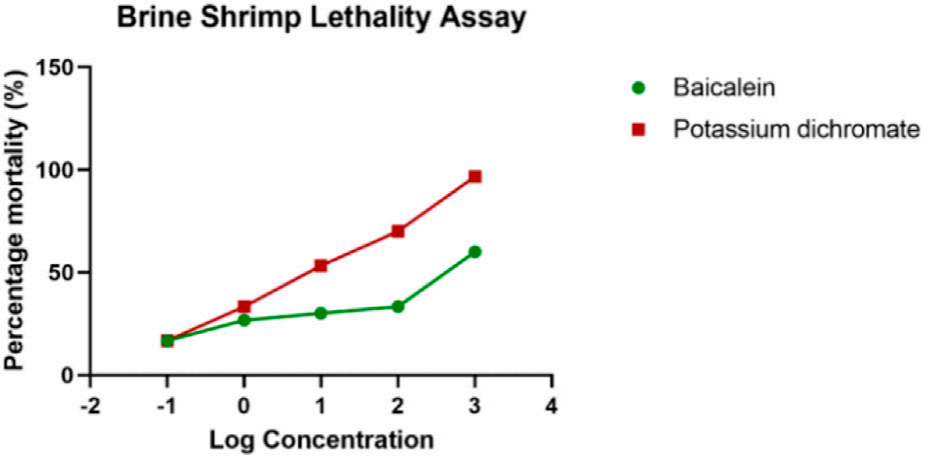
Brine shrimp lethality assay for baicalein.

The brine shrimp larva, *Artemia salina* L. (Artemiidae), is an invertebrate used in alternative tests to detect the toxicity of chemical and natural compounds. The assay has been routinely used to test the toxicity of a wide range of plant products in the past 30 years (Figure 3). *Artemia salina*is the most researched *Artemia* species, accounting for almost 90% of studies involving *Artemia* as an experimental test organism. Based on the assay, in the control sets, almost all shrimps survived throughout the observed period (24 h). In the highest treated concentration (1,000 μg/ml), the shrimps began dying only after 12 h with complete shrimp lethality seen after 21 h. Complete mortality was observed in the positive control potassium dichromate with an LC_50_ value of 6.4 μg/ml which is cytotoxic (Table 1; Figure 4). Baicalein showed considerable brine shrimp toxicity with an LC_50_ value of 244 μg/ml after 24 h (Table 1; Figure 4). The increase in mortality seen was proportional to the increase in concentrations, which provided linearity in the dose-response relationship of every compound tested.

**TABLE 1:** Brine shrimp lethality assay for baicalein.

S.no	Concentration (µg/ml)	Percentage death of nauplii following 24 h of exposure	
		Baicalein	Potassium dichromate
1	0.1	16.67 ± 3.30	16.67 ± 3.30
2	1.0	26.67 ± 3.30	33.30 ± 3.30
3	10.0	30.00 ± 0.00	53.30 ± 3.30
4	100.0	43.30 ± 3.30	70.00 ± 0.00
5	1000.0	60.00 ± 5.70	96.67 ± 3.30
	Log LC_50_	2.39	0.81
	LC_50_	244.00	6.44

Values are presented as the mean ± SD (*n* = 3).

Based on Logarto Parra et al (Logarto Parra et al., 2001) correlation, baicalein which showsLC_50_ < 10 μg/ml, possesses 50% lethal dose (LD_50_) between 100 and 1,000 mg/kg; LC_50_ < 20 μg/ml possesses LD_50_ between 1,000 and 2500 mg/kg while LC_50_ > 25 μg/ml possesses LD_50_ between 2500 and 8,000 mg/kg (Aponte and Petrunich-Rutherford, 2019). Since the LC_50_ of baicaleinfallsin the range of LC_50_ > 25 μg/ml, the LD_50_ for baicalein is expected to be between 2500 and 8,000 mg/kg. In the present study, baicalein which has LC_50_ values <1,000 μg/ml indicates its good biological activity.

### 3.2 Novel tank diving test

**FIGURE 5:**
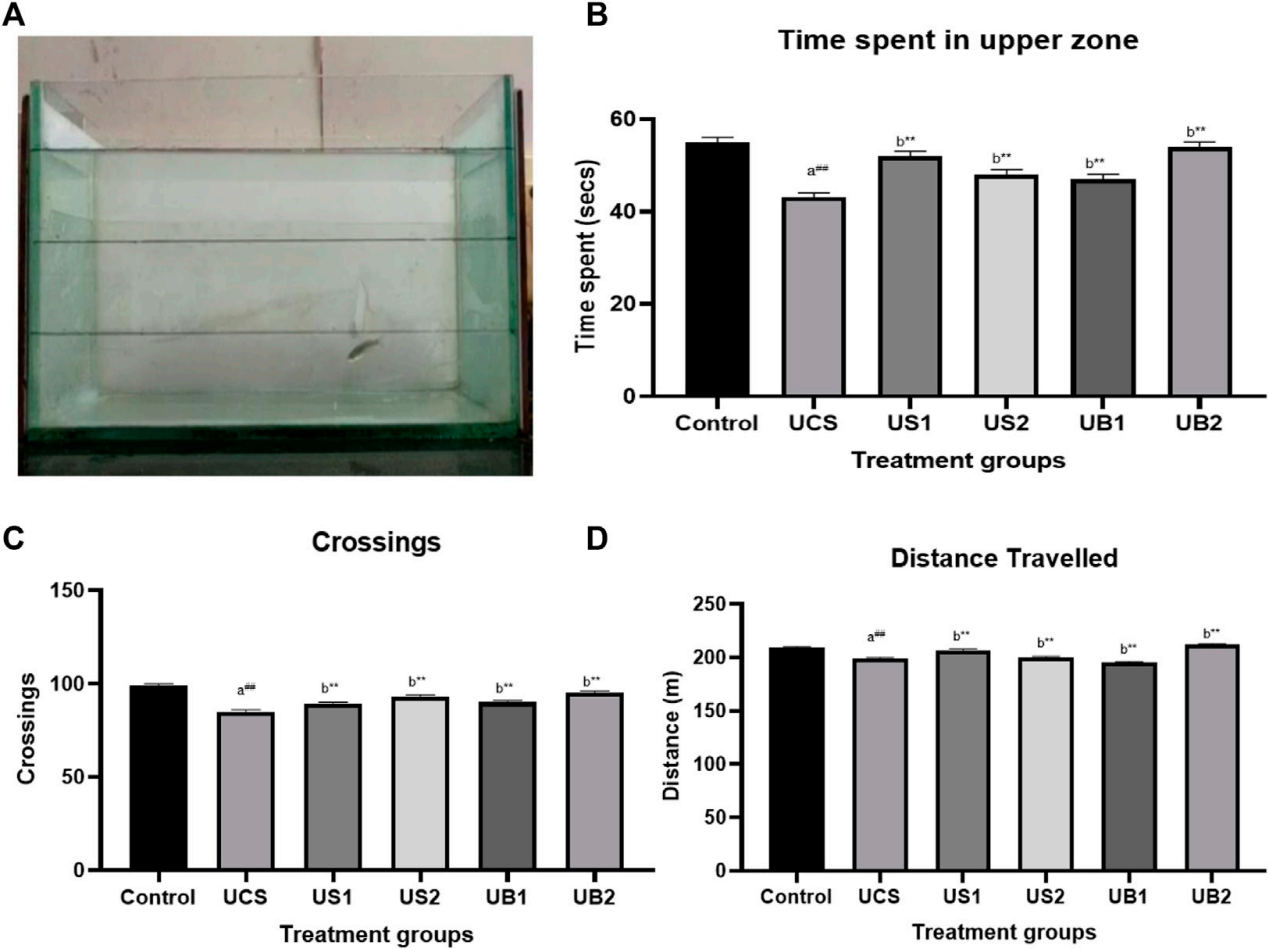
**(A)** Novel tank test of drug-treated group showing the exploratory behaviour of zebrafish (UCS) **(B)** The effect of treatment on time spent in the upper level of the tank in novel tank (UCS) **(C)** The effect of treatment on crossing of zones in novel tank (UCS) **(D)** The **e**ffect of treatment on total distance travelled in novel tank (UCS).Values were expressed as mean ± S.E.M. A one-way ANOVA followed by Bonferroni post hoc test (*n* = 8–10) was used. **(A)** Comparisons were made between the stressed group (UCS) with normal control. **(B)** Comparisons were made between US1, US2, UB1, and UB2 with stressed group (UCS). p-value ***represents *p* < 0.001, **represents *p* < 0.01, *represents *p* < 0.05, ^**##**^represents *p* < 0.01. **(B)** shows the influence of baicalein, diazepam and fluoxetine on behavioral parameters in zebrafish subjected to UCS. As expected, UCS increased the time spent in the bottom area and decreased the entries as well as the time in the top area. The control group (control) showed the time spent in the upper zone (55 s) and the stressed group (UCS) showed decreased in the time spent in the upper zone (42 s). Baicalein-treated groups (UB1 andUB2) were found to explore more in the upper levels of the tank following transfer to a novel tank in comparison to standard drugs. On the other hand, UB1 group has increased time spent (47 s) while the UB2 group had 54 s as the time spent [panel **(B)**]. Fluoxetine and diazepam-treated group exhibited increased in the time spent in the upper levels (at 48 s and 52 s respectively). The total distance travelled [panel **(C)**] was not significantly affected by the unpredictable chronic stress model but the number of crossing [panel **(D)**] was decreased by the UCS protocol.

**FIGURE 6:**
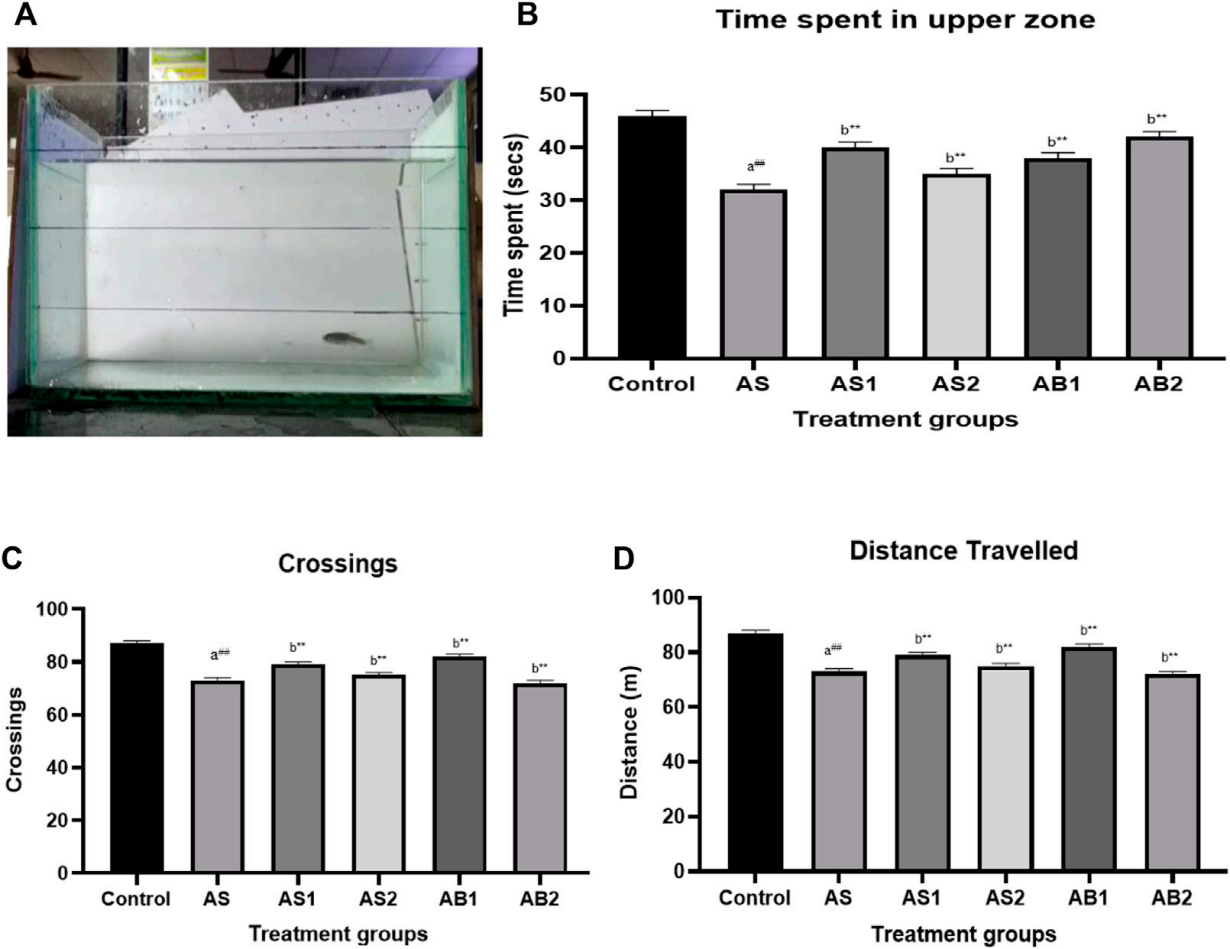
**(A)** Novel tank test of drug-treated group showing the exploratory behaviour of zebrafish (AS) **(B)** The effect of treatment on time spent in the upper level of the tank in novel tank (AS) **(C)** The effect of treatment on crossing of zones in novel tank (AS) **(D)** The effect of treatment on total distance travelled in novel tank (AS). Values are presented as the mean ± S.E.M. A one-way ANOVA followed by Bonferroni post hoc test (*n* = 8–10) was used. **(A)** Comparisons were made between the stressed group (AS) with the normal control. **(B)** Comparisons were made between AS1, AS2, AB1 andAB2 with the stressed group (AS). p-value ***represents *p* < 0.001, **represents *p* < 0.01, *represents *p* < 0.05, ^**##**^represents *p* < 0.01. **(B)** shows the effects of Baicalein (AB1 andAB2), diazepam (AS1) and fluoxetine (AS2) in zebrafishes involved in the acute stress model. As expected, diazepam significantly decreased the time spent in the bottom and increased the time spent the upper zone of the tank [panel **(B)**]. Baicalein-treated groups such as AB2 and AB2 significantly increase the time spent in the upper zone (38 and 42 s respectively). The distance travelled [panel **(D)**], the number of crossings [panel **(C)**] and entries to the bottom area was not affected by any intervention.

To simulate a more realistic environment when assessing the possible use of baicalein in patients with stress-related mental illnesses, the zebrafish was subjected to UCS for 7 days prior to treatment. The total distance travelled and crossings were utilised as locomotor activity indicators in the NTT. The ratio of time spent in the bottom region to the time spent in the top area is employed as a proxy for anxious behaviour in rodents, which corresponds to thigmotaxis in the open-field test. As expected, our procedure enhanced anxiety-like behaviour, as shown by the longer time spent in the bottom zone of the tank and fewer entry as well as time spent in the upper zone (Figures 5, 6).

### 3.3 Light/dark test

**FIGURE 7:**
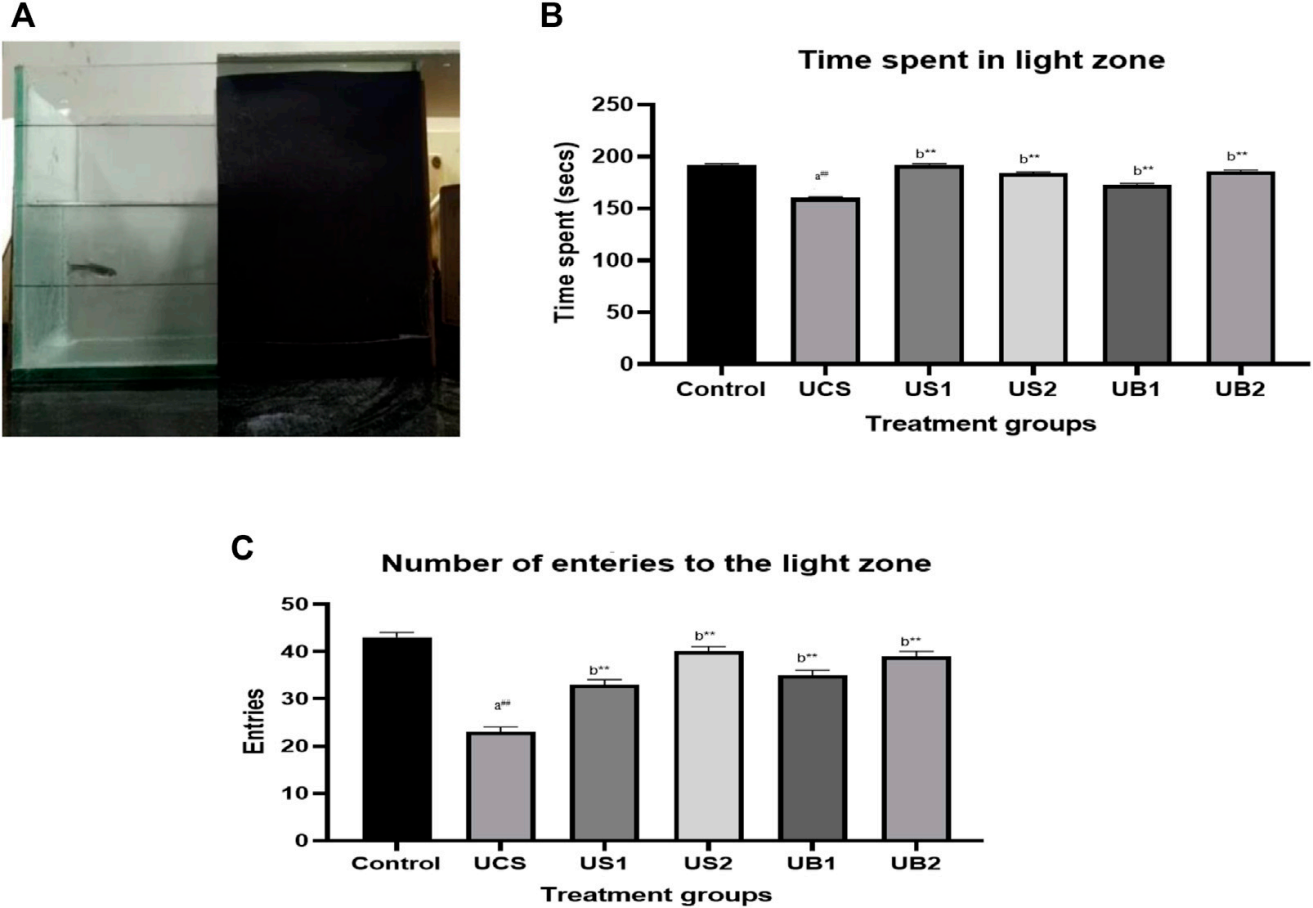
**(A)** Light dark test of drug-treated group of zebrafish (UCS) **(B)** The effect of treatment on the time spent in the light zone of the tank (UCS) **(C)** The effect of treatment on entries to the light zone of the tank (UCS). The data are presented as the mean ± S.E.M. A one-way ANOVA followed by a Bonferroni post hoc test was used (*n* = 8–10). **(A)** Comparisons were made between the stressed group (UCS) with the normal control. **(B)** Comparisons were made between US1, US2, UB1 and UB2 with the stressed group (UCS). p-value ***represents *p* < 0.001, **represents *p* < 0.01, *represents *p* < 0.05, ^**##**^represents *p* < 0.01.

In the light/dark task, the control group spent 192 s of 300 s in the light compartment which was divided into light and dark compartments (Figure 7B. On the other hand, the stressed group (UCS) spent 160 s in the light side indicating that the UCS model produced stress in zebrafish. In the treatment group, the baicalein-treated groups (UB1 and UB2) spent more time in the light zone for 173 s and 186 s respectively in comparison to the standard drugs such as diazepam (US1) and fluoxetine-treated (US2) group which spent more time in the light side of the tank for 192 s and 184 s respectively.

The stressed group (UCS) showed decreased entries to the light zone, compared to the control group which showed 23 entries. Both baicalein-treated group showed increase in the number of entries to the light side of the tank indicating the anxiety-alleviating property of baicalein following exposure to chronic stress as compared to the standard drugs (diazepam and fluoxetine) during the evaluation period. Baicalein-treated groups (UB1 and UB2) showed 35 and 39 entries respectively which were similar to the standard drugs [diazepam (US1) = 33 and fluoxetine (US2) = 40] [Figure 7C).

**FIGURE 8:**
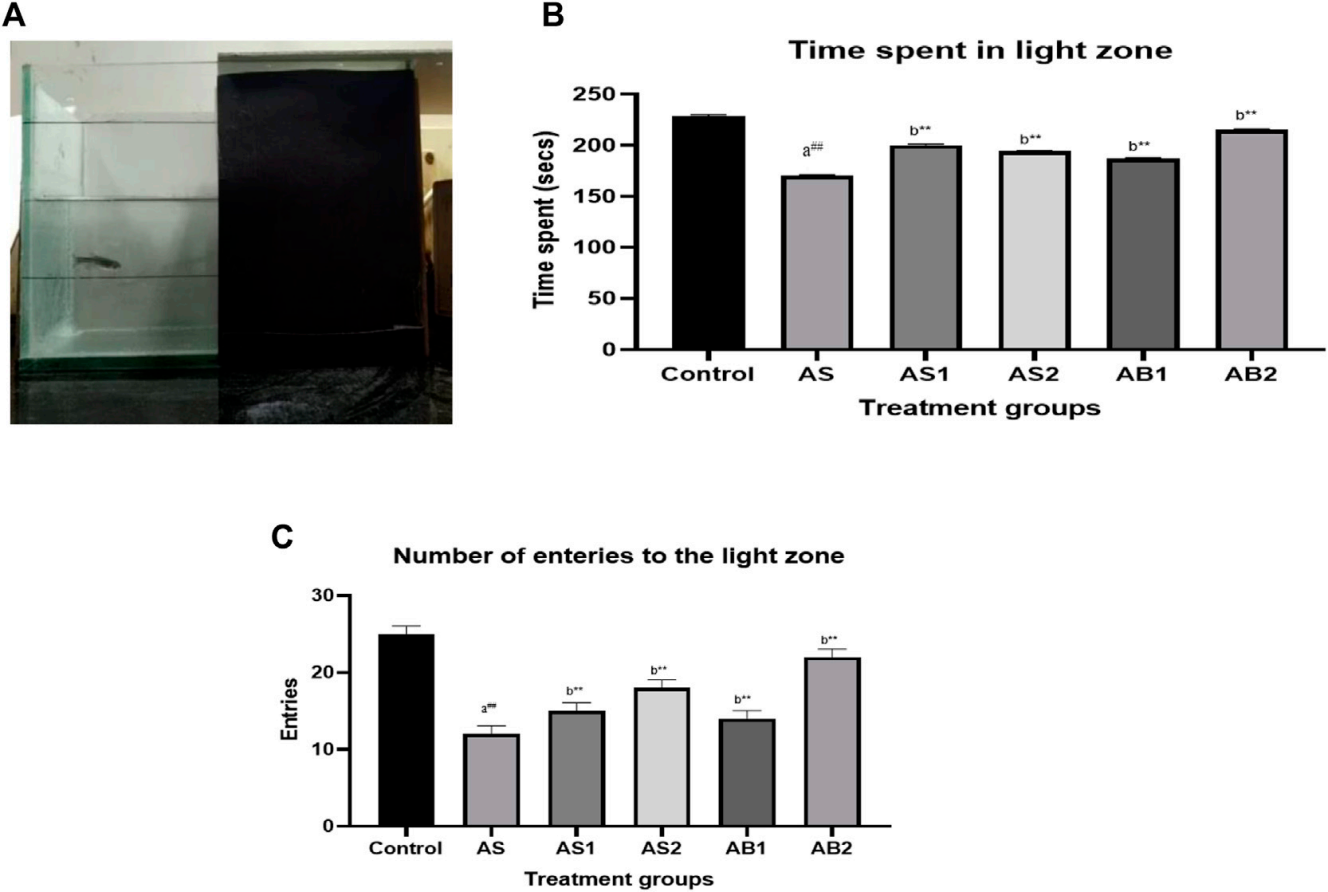
**(A)** Light dark test of drug-treated group of zebrafish (AS) **(B)** The effect of treatment on time spent in the light zone of the tank (AS) **(C)** The effect of treatment on entries to the light zone of the tank (AS). Values are presented as the mean ± S.E.M. A one-way ANOVA followed by Bonferroni post hoc test was used (*n* = 8–10). **(A)** Comparisons were made between the Stressed group (AS) with normal control. **(B)** Comparisons were made between AS1, AS2, and AB1 andAB2 with the stressed group (AS). p-value ***represents *p* < 0.001, **represents *p* < 0.01, *represents *p* < 0.05, ^**##**^represents *p* < 0.01, ns represents being not significant.

In an acute stress model, the control group spent 229 s of 300 s in the light compartment (Figure 8). The acute stressed group (AS) spent 170 s in the light side indicating that the acute stress model did elicit some stress in the zebrafish. In the treatment group, the baicalein-treated groups (AB1 andAB2) spent more time in the light zone (187 s and 215 s respectively) in comparison to the standard drugs, such as diazepam (AS1) and fluoxetine-treated (AS2) groups which spent more time in the light side of the tank.

The acute stress group (AS) showed decreased entries to the light zone (12 compared to the control group which showed 25 entries). Both baicalein-treated groups showed an increase in the number of entries to the light side of the tank indicating the anxiety-alleviating property of baicalein following exposure to acute stress as compared to the standard drugs diazepam and fluoxetine during the evaluation period. Baicalein-treated groups (AB1 and AB2) showed 15 and 18 entries respectively which was similar to that of the standard drugs [diazepam (AS1) = 15 and fluoxetine (AS2) = 18] (Figure 8).

### 3.4 *In silico* docking analysis

Baicalein and the known active drugs for respective targets were docked (Tables 2, 3). Baicalein shows a docking score of -8.272 for GABA(A) receptor which is greater than the docking score of standard drugs diazepam and alprazolam.

**TABLE 2:** Docking analysis of ligands against GABA(A) Receptor (PDB ID: 6X3X).

Compounds	PubChem CID	Dock score	E-model score
Baicalein	5281605	−8.272	−43.791
Diazepam	3016	−6.637	−33.205
Alprazolam	2118	−7.833	−73.019

**TABLE 3:** Docking analysis of ligands against SERT (PDB ID: 5I73).

Compounds	PubChem CID	Dock score	E-model score
Baicalein	5281605	−2.772	−24.791
Citalopram	2771	−3.218	−25.737
Fluoxetine	3386	−3.84	−25.522

**FIGURE 9:**
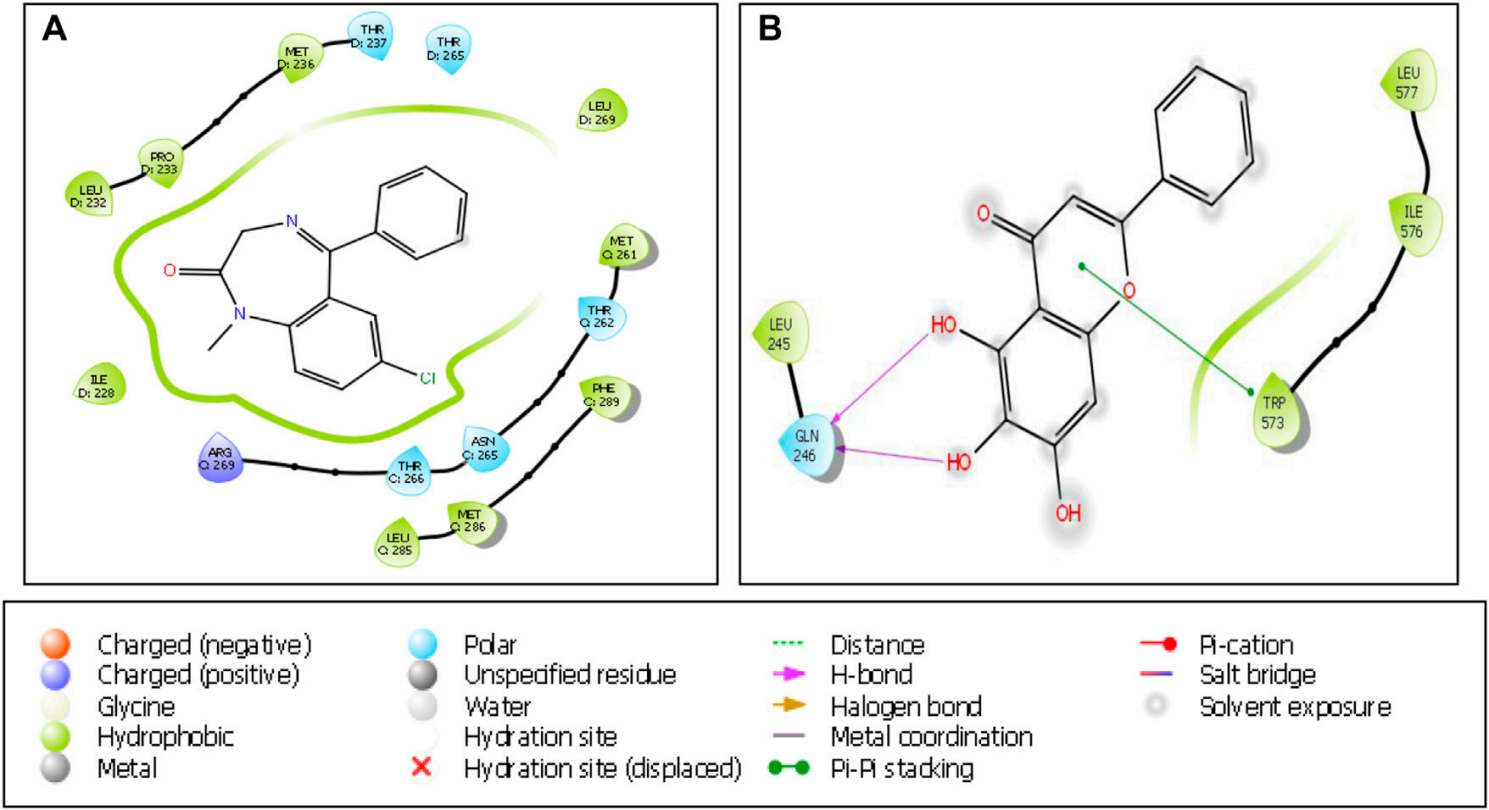
Binding interactions of **(A)** baicalein with GABA**(A)**receptor (PDB ID: 6X3X) and **(B)** baicalein with serotonin transporter (PDB ID: 5I73).

For serotonin transporter, fluoxetine showed a higher affinity (docking score -3.84) followed by citalopram (docking score -3.218) and baicalein (docking score -2.772). The binding interaction of baicalein with GABA(A) and SERT are seen in Figures 9A,B.

## 4 Discussion

To the best of our knowledge, this is the first study to illustrate the preventative effect of baicalein on zebrafish stress responses. Plant sources and isolated phytochemicals have a variety of pharmacological effects on brain-related illnesses such as anxiety, depression and cognitive deficits and have piqued interest in the creation of therapeutic medicines. Flavonoids are polyphenolic phytochemicals found in practically all plants, fruits, flowers, seeds and vegetables. In rat models, a number of key flavonoids extracted from fruits and plants have potential preclinical impacts on emotional disorders such as sadness and anxiety.

Several flavonoids have been discovered to have anti-anxiety and anti-depressive effects in the brain by exerting diverse pathways (Ko et al., 2020). In 1998, Liao *et al* (Liao et al., 1998) reported that three flavones have affinity to benzodiazepine binding site including baicalein, oroxylin A and skullcap flavone II by using benzodiazepine binding assay (Liao et al., 1998). In a Vogel conflict test adapted for ICR mice in 2003, similar group of researchers investigated whether baicalein and its 7-glucuronide, baicalein, have anxiolytic-like effects. They concluded that the anxiolytic-like effect of baicalein or baicalin may be mediated through activation of the benzodiazepine binding site of GABA_A_ receptors (Liao et al., 2003).

De Carvalho et al. (de Carvalho et al., 2011) conducted one such study to assess the anxiolytic-like and related properties of baicalein after central administration (i.c.v.) in mice and concluded that baicalein promotes anxiolytic-like and sedative effects as well as pharmacological activities dependent on GABAergic non-benzodiazepine sites but not on the 5-HT system (de Carvalho et al., 2011). The modification of the connection between tropomyosin receptor kinase (TrkB) and the GABA_A_ receptor β (GABA_A_Rβ), as well as the increase in synaptic protein expression, may be responsible for baicalein’s neuroprotective effects. Baicalein was described as a unique synaptoprotective approach for the treatment of mild hepatic encephalopathy in one of the earlier study (Ding et al., 2018). Additionally, they have shown that baicalein modulates GABA_A_R to trigger the TrkB/AKT/synapse-related protein pathway. In mild hepatic encephalopathy rats, baicalein restores DA-induced long-term potentiation impairment *via* promoting the activation of GABA_A_R (Ding et al., 2018). On an ischemia/reperfusion gerbil model, Dai *et al* (Dai et al., 2013) discovered that baicalin significantly elevated the expression of GABA_A_Rα1 and γ2 subunits at the mRNA and protein levels in the hippocampal CA1 subfield. In ischemic gerbils treated with baicalin, the protein levels of KCC2 (K^+^-Cl^-^) and NKCC1 (Na^+^-K^+^-Cl^-^) both changed concurrently. According to these results, baicalin’s neuroprotective effects on ischemia-induced neuronal damage in gerbils are linked to GABA_A_R-mediated inhibitory responses (Dai et al., 2013). Positive allosteric modulators of the benzodiazepine site and/or non-benzodiazepine site of the GABA_A_ receptor include baicalin and its aglycone baicalein (Hui et al., 2000). Another study confirmed the anxiolytic-like effects of baicalein in the elevated plus maze and the Vogel conflict test, and it revealed that baicalein’s pharmacological activity involved GABA_A_ receptors (Xu et al., 2006). Baicalein may prevent GABA_A_R suppression induced by D1R stimulation (Xu et al., 2006).

In our research, the anti-anxiety mechanism of baicalein is predicted using molecular docking studies against various targets, in order to provide extra body of evidence supporting the clinical evaluation of baicalein in chronic and acute stress induced anxiety in various zebrafish models. In the molecular docking study, we chose GABA_A_ receptor which is the target for the benzodiazepine class of drugs such as diazepam, chlordiazepoxide, clonazepam, and serotonin transporter (SERT) which is the target for various SSRI such as fluoxetine and citalopram (Liao et al., 1998). Baicalein has a better affinity towards GABA_A_ receptor as compared to standard drugs such as diazepam while incurring less affinity towards SERT. Mechanism based docking studies were performed and it was in correlation with previous baicalein based literatures, in future brain neurotransmitters estimation studies will be carried out in zebrafish for mechanism based identification along with the standard drugs. Overall, our findings confirm that the anti-anxiety activity of baicalein may be due to its interaction with GABA_A_ receptor as predicted by some previous researchers (Liao et al., 1998; Liao et al., 2003).

The ability of non-human animals (such as zebrafish) to be subjected to experimental, genetic and pharmacological treatments is a significant advantage for modelling brain disorders. Furthermore, zebrafish behavioural traits, genetic variables and pharmacological sensitivity are frequently similar to those reported in rat models of brain diseases and clinical populations. The zebrafish model lends itself well to high-throughput pharmacological screening for anxiolytics. The zebrafish is swiftly becoming a popular model organism for studying stress-induced alterations in early life behaviours and brain circuitry (Eachus et al., 2021). The connectome, which reflects the evolution of the brain’s highly organised connection matrix, offers the opportunity of elucidating the pathophysiology of anxiety disease, and the zebrafish brain is an ideal subject to explore its connections (Ma et al., 2020). The anxiety-related behavioural tests in zebrafsh are helpful in understanding anxiety disorders in mammals, including humans; for example, agitated zebrafsh avoid the centre of an open field, which is similar to centre avoidance in humans with high anxiety sensitivity (Blaser and Rosemberg, 2012). In the novel tank test (Stewart et al., 2011), measurements of anxiety in adult fish include a latency to explore the top or a stronger tendency to remain at the bottom. The fish are free to explore brightly lit and dark arenas in the light–dark test, but when the zebrafish spend more time in the dark (scototaxis), it is an anxiety-like reaction which can be 2modified bidirectionally by anxiolytic or anxiogenic therapies (Park et al., 2016).

**FIGURE 10:**
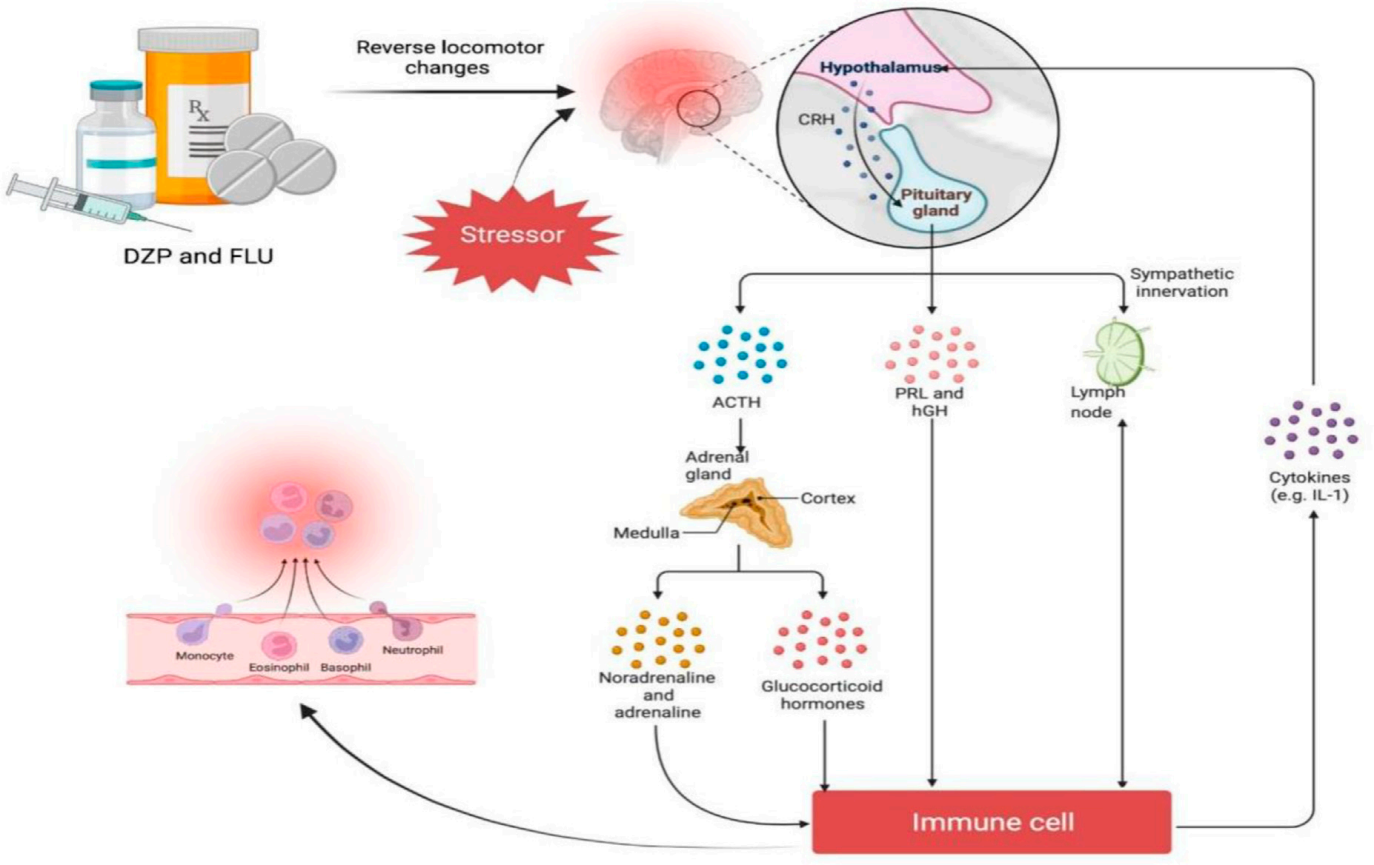
The acute stress protocol induced locomotor changes that were reversed by diazepam (DZP) and fluoxetine treatments (FLU). The hypothalamic-pituitary-adrenal (HPA) axis results in a rise of corticosteroids levels in the blood, which are subsequently delivered to the spleen and periphery, where they decrease a number of immune processes. FLU and DZP both can to act as anxiolytics by decreasing cortisol responses to acute stress. Abbreviations: ACTH, Adrenocorticotropin; PRL, Prolactin; hGH, human growth hormone; IL-1, Interleukin-1.

The result of the acute stress models indicates that acute stress increase anxiety as seen in the behaviour of the zebrafish. The increased time spent in the bottom area and the decreased exploration to the upper area in the stressed models indicates the presence of anxiety. Acute administration of diazepam and fluoxetine to acute stress models reduced the time spent in the bottom area, indicating that the behavioural alterations induced by the acute stress protocol have been reversed. FLU and DZP reversed the locomotor alterations generated by the acute stress treatment (Figure 10). These agents did not cause drowsiness or meaningful motor side effects at the concentrations used. As previously demonstrated in zebrafish (Abreu et al., 2014), the anxiolytic effects of FLU and DZP may be attributed to the blocking of cortisol responses to acute stress. In fact, some investigations have reported that FLU has an effect on the stress neuroendocrine axis. FLU affects the genetic expression of glucocorticoid and mineralocorticoid receptors, as well as the expression of GABA transporters in the brain, leading to reduction in the stress response. FLU has an anxiolytic effect on neuropeptides and neurosteroids in addition to regulating serotonin (Wong et al., 2010; Adzic et al., 2013). Additionally, studies using light/dark and new tank tests have established DZP’s anxiolytic impact in zebrafish (Gebauer et al., 2011; Levin, 2011).

The acute administration of baicalein in acute stress induced anxiety models significantly decreased the time spent in the bottom of the tank similar to that seen in standard drugs indicating baicalein’s anxiolytic effect against acute stress. The acute stress models indicated a decreased time spent in the light zone indicating that the acute stress induced anxiolytic behaviours and the standard drugs increased the time spent in the light zone in the light/dark test, a protocol that has been pharmacologically validated with benzodiazepines, buspirone and fluoxetine (Gebauer et al., 2011; Maximino et al., 2013). UCS zebrafish model increases the anxiety level in zebrafish. The current UCS technique was confirmed by Piato et al. based on the Group Behavior Task (GBT), which entails analysing animals' movement, colour, shoal cohesiveness and height on the water column simultaneously in a 2.7 L tank 24 × 8 × 20 cm (length×width×height) with 15 cm of water level. The UCS procedure in zebrafish is a good alternative to other animal models for researching the neurobiology and the effects of chronic stress since it has a superior cost-benefit ratio. After 7 days of stress, the model appears to have a strong construct validity (the same neurological basis as rodents and humans) for anxiety and possibly for depression as well (Piato et al., 2011). The total distance travelled and crossings were utilized as locomotor activity indicators in the NTT. The ratio of the time spent in the bottom region to the time spent in the top area is employed as a proxy for anxious behaviour in rodents, which corresponds to thigmotaxis in the open-field test.

As expected, our procedure enhances anxiety-like behaviour, as indicated by the longer time spent in the bottom zone of the tank and fewer entry as well as time spent in the upper zone. Baicalein had no influence on locomotor activity in non-stressed mice as well as in stressed animals. Different anxiolytics such as bromazepam, diazepam, buspirone and fluoxetine, had similar effects in the new tank test (Marcon et al., 2016). Animal handling was conducted in a consistent manner to eliminate the potential that the UCS group become less anxious on the test day than the never-handled control group. Stressors were also applied twice daily in a randomised, unpredictable manner to avoid habituation in stressed groups. These findings indicate baicalein’s anti-anxiety action in the treatment of acute and chronic stress-induced anxiety. The study demonstrates that behavioural investigations in zebrafish models have a considerable utility that is comparable to rodent models. As a result, the novel tank diving test and the light/dark model may become standard models for assessing anxiety in zebrafish for preclinical studies.

Due to their genetic and physiological parallels to the human system, rats have been chosen as anxiety models. Zebrafish (*Danio rerio*) have a genetic code that is identical to human beings and in fact, share up to 70% of the human genes. Additionally, it is estimated that 84% of the genes associated with human disease have a zebrafish counterpart (Crouzier et al., 2021). In order to improve our understanding of brain development, dysfunction and their genetic and pharmacological regulation, both larval and adult zebrafish were used. It is inexpensive and delivers results that are more accurate. The behavioural measures are beneficial for anxiety assessment in zebrafish because of their simplicity and ability to detect distinct and common behavioural changes with different anxiolytic drugs.

## 5 Conclusion

The neuropharmacological action of baicalein on stress-induced anxiety is confirmed as evidenced from the behavioral parameters evaluated following exposure to stress. Baicalein is a promising therapy for the treatment of stress and related psychiatric illnesses since it ameliorates the effects induced by UCS and acute stress. We have chosen the GABA_A_ receptor as the target to examine and prove mechanistically in the molecular docking investigation. Mechanism-based docking research were carried out, and they were in agreement with earlier baicalein-based literature’s; in the future, brain neurotransmitter estimating studies in zebrafish model need to be carried out for mechanism-based identification with standard drugs. Despite its high efficacy and safety, baicalein has not yet received regulatory approval as a therapeutic drug, and scientific investigation into its potential as an effective anti-anxiety treatment is still in its early stages. Therefore, more research is still needed to fully understand the effectiveness and potential therapeutic benefits of baicalein in the treatment of psychiatric illnesses such as anxiety. Although the results available so far is encouraging, the molecular basis and levels of expression of numerous synthetic enzymes and transporter genes, which are necessary to produce anti-anxiety effects, still need to be discovered through future investigations. To prove baicalein’s effectiveness in treating anxiety disorders in humans, clinical trials are also needed.

## Data Availability

The datasets presented in this study can be found in online repositories. The names of the repository/repositories and accession number(s) can be found in the article/supplementary material.

## References

[B1] Abreu M. S. de Koakoski G. Ferreira D. Oliveira T. A. Rosa J. G. S. da Gusso D. (2014). Diazepam and fluoxetine decrease the stress response in zebrafish. PLoS One 9 (7), e103232. 10.1371/journal.pone.0103232 PubMed Abstract | 10.1371/journal.pone.0103232 | Google Scholar 25054216 PMC4108411

[B2] Adzic M. Lukic I. Mitic M. Djordjevic J. Elaković I. Djordjevic A. (2013). Brain region- and sex-specific modulation of mitochondrial glucocorticoid receptor phosphorylation in fluoxetine treated stressed rats: Effects on energy metabolism. Psychoneuroendocrinology 38 (12), 2914–2924. 10.1016/j.psyneuen.2013.07.019 PubMed Abstract | 10.1016/j.psyneuen.2013.07.019 | Google Scholar 23969420

[B3] Ali S. van Mil H. G. Richardson M. K. (2011). Large-scale assessment of the zebrafish embryo as a possible predictive model in toxicity testing. PLoS ONE 6 (6), e21076. 10.1371/journal.pone.0021076 PubMed Abstract | 10.1371/journal.pone.0021076 | Google Scholar 21738604 PMC3125172

[B4] Aponte Amy Petrunich-Rutherford Maureen L. (2019). Acute net stress of young adult zebrafish (*Danio rerio*) is not sufficient to increase anxiety-like behavior and whole-body cortisol. PeerJ 7, e7469. 10.7717/peerj.7469 PubMed Abstract | 10.7717/peerj.7469 | Google Scholar 31410315 PMC6689218

[B5] Blaser R. E. Rosemberg D. B. (2012). Measures of anxiety in zebrafish (*Danio rerio*): Dissociation of black/white preference and novel tank test. PLoS One 7 (5), e36931. 10.1371/journal.pone.0036931 PubMed Abstract | 10.1371/journal.pone.0036931 | Google Scholar 22615849 PMC3355173

[B6] Brinza I. Ayoub I. M. Eldahshan O. A. Hritcu L. (2021). Baicalein 5, 6-dimethyl ether prevents memory deficits in the scopolamine zebrafish model by regulating cholinergic and antioxidant systems. Plants 10 (6), 1245. 10.3390/plants10061245 PubMed Abstract | 10.3390/plants10061245 | Google Scholar 34207381 PMC8233988

[B7] Bystritsky A. Khalsa S. S. Cameron M. E. Schiffman J. (2013). Current diagnosis and treatment of anxiety disorders. P Trans. 38 (1), 30–57. PubMed Abstract | Google Scholar PMC3628173 23599668

[B8] Caio M. Daniele L. M. Barbara D. Mezzomo N. J. Stefanello F. V. de S Prestes A. (2018). Extending the analysis of zebrafish behavioral endophenotypes for modeling psychiatric disorders: Fear conditioning to conspecific alarm response. Behav. Process. 149, 35–42. 10.1016/j.beproc.2018.01.020 10.1016/j.beproc.2018.01.020 | Google Scholar 29409977

[B9] Cheng R. K. Jesuthasan S. J. Penney T. B. (2014). Zebrafish forebrain and temporal conditioning. Philos. Trans. R. Soc. Lond. B Biol. Sci. 369 (1637), 20120462. 10.1098/rstb.2012.0462 PubMed Abstract | 10.1098/rstb.2012.0462 | Google Scholar 24446496 PMC3895987

[B10] Chrousos G. P. (2009). Stress and disorders of the stress system. Nat. Rev. Endocrinol. 5, 374–381. 10.1038/nrendo.2009.106 PubMed Abstract | 10.1038/nrendo.2009.106 | Google Scholar 19488073

[B11] Crouzier L. Richard E. M. Sourbron J. Lagae L. Maurice T. Delprat B. (2021). Use of zebrafish models to boost research in rare genetic diseases. Int. J. Mol. Sci. 22, 13356. 10.3390/ijms222413356 10.3390/ijms222413356 | Google Scholar 34948153 PMC8706563

[B12] Dai J. Chen L. Qiu Y. M. Li S. Q. Xiong W. H. Yin Y. H. (2013). Activations of GABAergic signaling, HSP70 and MAPK cascades are involved in baicalin's neuroprotection against gerbil global ischemia/reperfusion injury. Brain Res. Bull. 90, 1–9. 10.1016/j.brainresbull.2012.09.014 PubMed Abstract | 10.1016/j.brainresbull.2012.09.014 | Google Scholar 23041106

[B13] de Abreu M. S. Demin K. A. Giacomini A. C. V. V. Amstislavskaya T. G. Strekalova T. Maslov G. O. (2021). Understanding how stress responses and stress-related behaviors have evolved in zebrafish and mammals. Neurobiol. Stress 15, 100405. 10.1016/j.ynstr.2021.100405 10.1016/j.ynstr.2021.100405 | Google Scholar 34722834 PMC8536782

[B14] de Carvalho R. S. M. Duarte F. S. de Lima T. C. M. (2011). Involvement of GABAergic non-benzodiazepine sites in the anxiolytic-like and sedative effects of the flavonoid baicalein in mice. Behav. Brain Res. 221 (1), 75–82. 10.1016/j.bbr.2011.02.038 PubMed Abstract | 10.1016/j.bbr.2011.02.038 | Google Scholar 21377498

[B15] Déciga-Campos M. Rivero-Cruz I. Arriaga-Alba M. Castañeda-Corral G. Angeles-López G. E. Navarrete A. (2007). Acute toxicity and mutagenic activity of Mexican plants used in traditional medicine. J. Ethnopharmacol. 110 (2), 334–342. 10.1016/j.jep.2006.10.001 PubMed Abstract | 10.1016/j.jep.2006.10.001 | Google Scholar 17101253

[B16] Ding S. Zhuge W. Hu J. Yang J. Wang X. Wen F. (2018). Baicalin reverses the impairment of synaptogenesis induced by dopamine burden via the stimulation of GABA_A_R-TrkB interaction in minimal hepatic encephalopathy. Psychopharmacol. Berl. 235 (4), 1163–1178. 10.1007/s00213-018-4833-8 PubMed Abstract | 10.1007/s00213-018-4833-8 | Google Scholar PMC5869945 29404643

[B17] Duman R. S. Aghajanian G. K. Sanacora G. Krystal J. H. (2016). Synaptic plasticity and depression: New insights from stress and rapid-acting antidepressants. Nat. Med. 22, 238–249. 10.1038/nm.4050 PubMed Abstract | 10.1038/nm.4050 | Google Scholar 26937618 PMC5405628

[B18] Eachus H. Choi M. K. Ryu S. (2021). The effects of early life stress on the brain and behaviour: Insights from zebrafish models. Front. Cell Dev. Biol. 9, 657591. 10.3389/fcell.2021.657591 PubMed Abstract | 10.3389/fcell.2021.657591 | Google Scholar 34368117 PMC8335398

[B19] Egan R. J. Bergner C. L. Hart P. C. Cachat J. M. Canavello P. R. Elegante M. F. (2009). Understanding behavioral and physiological phenotypes of stress and anxiety in zebrafish. Behav. Brain Res. 205 (1), 38–44. 10.1016/j.bbr.2009.06.022 PubMed Abstract | 10.1016/j.bbr.2009.06.022 | Google Scholar 19540270 PMC2922906

[B20] Gebauer D. L. Pagnussat N. Piato A. L. Schaefer I. C. Bonan C. D. Lara D. R. (2011). Effects of anxiolytics in zebrafish: Similarities and differences between benzodiazepines, buspirone and ethanol. Pharmacol. Biochem. Behav. 99 (3), 480–486. 10.1016/j.pbb.2011.04.021 PubMed Abstract | 10.1016/j.pbb.2011.04.021 | Google Scholar 21570997

[B21] Horzmann K. A. Freeman J. L. (2016). Zebrafish get connected: Investigating neurotransmission targets and alterations in chemical toxicity. Toxics 4 (3), 19. 10.3390/toxics4030019 PubMed Abstract | 10.3390/toxics4030019 | Google Scholar 28730152 PMC5515482

[B22] Hui K. M. Wang X. H. Xue H. (2000). Interaction of flavones from the roots of *Scutellaria baicalensis* with the benzodiazepine site. Planta Med. 66 (1), 91–93. 10.1055/s-0029-1243121 PubMed Abstract | 10.1055/s-0029-1243121 | Google Scholar 10705749

[B23] Ibrahim M. Mussulini B. H. M. Moro L. de Assis A. M. Rosemberg D. B. de Oliveira D. L. (2014). Anxiolytic effects of diphenyl diselenide on adult zebrafish in a novelty paradigm. Prog. Neuropsychopharmacol. Biol. Psychiatry 54, 187–194. 10.1016/j.pnpbp.2014.06.002 PubMed Abstract | 10.1016/j.pnpbp.2014.06.002 | Google Scholar 24936773

[B24] Jiang X. Zhou J. Lin Q. Gong G. Sun H. Liu W. (2018). Anti-angiogenic and anticancer effects of baicalein derivatives based on transgenic zebrafish model. Bioorg. Med. Chem. 26 (15), 4481–4492. 10.1016/j.bmc.2018.07.037 PubMed Abstract | 10.1016/j.bmc.2018.07.037 | Google Scholar 30098912

[B25] Kalueff A. V. Stewart A. M. Gerlai R. (2014). Zebrafish as an emerging model for studying complex brain disorders. Trends Pharmacol. Sci. 35 (2), 63–75. 10.1016/j.tips.2013.12.002 PubMed Abstract | 10.1016/j.tips.2013.12.002 | Google Scholar 24412421 PMC3913794

[B26] Khan K. M. Collier A. D. Meshalkina D. A. Kysil E. V. Khatsko S. L. Kolesnikova T. (2017). Zebrafish models in neuropsychopharmacology and CNS drug discovery. Br. J. Pharmacol. 174 (13), 1925–1944. 10.1111/bph.13754 PubMed Abstract | 10.1111/bph.13754 | Google Scholar 28217866 PMC5466539

[B27] Ko Y. H. Kim S. K. Lee S. Y. Jang C. G. (2020). Flavonoids as therapeutic candidates for emotional disorders such as anxiety and depression. Arch. Pharm. Res. 43 (11), 1128–1143. 10.1007/s12272-020-01292-5 PubMed Abstract | 10.1007/s12272-020-01292-5 | Google Scholar 33225387

[B28] Levin E. D. Bencan Z. Cerutti D. T. (2007). Anxiolytic effects of nicotine in zebrafish. Physiol. Behav. 90 (1), 54–58. 10.1016/j.physbeh.2006.08.026 PubMed Abstract | 10.1016/j.physbeh.2006.08.026 | Google Scholar 17049956

[B29] Levin E. D. (2011). Zebrafish assessment of cognitive improvement and anxiolysis: Filling the gap between *in vitro* and rodent models for drug development. Rev. Neurosci. 22 (1), 75–84. 10.1515/RNS.2011.009 PubMed Abstract | 10.1515/RNS.2011.009 | Google Scholar 21615262 PMC4691346

[B30] Lezak K. R. Missig G. Carlezon W. A. Jr (2017). Behavioral methods to study anxiety in rodents. Dialogues Clin. Neurosci. 19 (2), 181–191. 10.31887/dcns.2017.19.2/wcarlezon PubMed Abstract | 10.31887/dcns.2017.19.2/wcarlezon | Google Scholar 28867942 PMC5573562

[B31] Liao J-F. Hung W-Y. Chen C-F. (2003). Anxiolytic-like effects of baicalein and baicalin in the Vogel conflict test in mice. Eur. J. Pharmacol. 464 (2–3), 141–146. 10.1016/s0014-2999(03)01422-5 PubMed Abstract | 10.1016/s0014-2999(03)01422-5 | Google Scholar 12620506

[B32] Liao J. F. Wang H. H. Chen M. C. Chen C. C. Chen C. F. (1998). Benzodiazepine binding site-interactive flavones from *Scutellaria baicalensis* root. Planta Med. 64 (6), 571–572. 10.1055/s-2006-957517 PubMed Abstract | 10.1055/s-2006-957517 | Google Scholar 9776664

[B33] Logarto Parra A. Silva Yhebra R. Guerra Sardiñas I. Iglesias Buela L. (2001). Comparative study of the assay of *Artemia salina* L. and the estimate of the medium lethal dose (LD50 value) in mice, to determine oral acute toxicity of plant extracts. Phytomedicine 8 (5), 395–400. 10.1078/0944-7113-00044 PubMed Abstract | 10.1078/0944-7113-00044 | Google Scholar 11695884

[B34] Ma M. Kler S. Pan Y. A. (2020). Structural neural connectivity analysis in zebrafish with restricted anterograde transneuronal viral labeling and quantitative brain mapping. Front. Neural Circuits 13, 85. 10.3389/fncir.2019.00085 PubMed Abstract | 10.3389/fncir.2019.00085 | Google Scholar 32038180 PMC6989443

[B35] Marcon M. Herrmann A. P. Mocelin R. Rambo C. L. Koakoski G. Abreu M. S. (2016). Prevention of unpredictable chronic stress-related phenomena in zebrafish exposed to bromazepam, fluoxetine and nortriptyline. Psychopharmacol. Berl. 233 (21–22), 3815–3824. 10.1007/s00213-016-4408-5 PubMed Abstract | 10.1007/s00213-016-4408-5 | Google Scholar 27562666

[B36] Maximino C. Puty B. Benzecry R. Araújo J. Lima M. G. de Jesus Oliveira Batista E. (2013). Role of serotonin in zebrafish (*Danio rerio*) anxiety: Relationship with serotonin levels and effect of buspirone, WAY 100635, SB 224289, fluoxetine and para-chlorophenylalanine (pCPA) in two behavioral models. Neuropharmacology 71, 83–97. 10.1016/j.neuropharm.2013.03.006 PubMed Abstract | 10.1016/j.neuropharm.2013.03.006 | Google Scholar 23541719

[B37] Miller A. H. Raison C. L. (2016). The role of inflammation in depression: From evolutionary imperative to modern treatment target. Nat. Rev. Immunol. 16, 22–34. 10.1038/nri.2015.5 PubMed Abstract | 10.1038/nri.2015.5 | Google Scholar 26711676 PMC5542678

[B38] Mocelin R. Marcon M. D'ambros S. Mattos J. Sachett A. Siebel A. M. (2019). N-acetylcysteine reverses anxiety and oxidative damage induced by unpredictable chronic stress in zebrafish. Mol. Neurobiol. 56 (2), 1188–1195. 10.1007/s12035-018-1165-y PubMed Abstract | 10.1007/s12035-018-1165-y | Google Scholar 29876880

[B49] Mocelin R. Herrmann A. P. Marcon M. Rambo C. L. Rohden A. Bevilaqua F. (2015). N-acetylcysteine prevents stress-induced anxiety behavior in zebrafish. Pharmacol. Biochem. Behav. 139, 121–126. 10.1016/j.pbb.2015.08.006 PubMed Abstract | 10.1016/j.pbb.2015.08.006 | Google Scholar 26261019

[B39] Muniandy Y. (2018). The use of larval zebrafish (*Danio rerio*) model for identifying new anxiolytic drugs from herbal medicine. Zebrafish 15 (4), 321–339. 10.1089/zeb.2018.1562 PubMed Abstract | 10.1089/zeb.2018.1562 | Google Scholar 29851363

[B40] Nachammai V. Jeyabalan S. Muthusamy S. (2021). Anxiolytic effects of silibinin and naringenin on zebrafish model: A preclinical study. Indian J. Pharmacol. 53 (6), 457–464. 10.4103/ijp.IJP_18_20 PubMed Abstract | 10.4103/ijp.IJP_18_20 | Google Scholar 34975133 PMC8764982

[B41] Nathana J. M. Barbara D. F. Duarte T. Quadros V. A. Canzian J. Pompermaier A. (2019). Taurine modulates the stress response in zebrafish. Horm. Behav. 109, 44–52. 10.1016/j.yhbeh.2019.02.006 PubMed Abstract | 10.1016/j.yhbeh.2019.02.006 | Google Scholar 30742830

[B42] Niedzielska E. Smaga I. Gawlik M. Moniczewski A. Stankowicz P. Pera J. (2016). Oxidative stress in neurodegenerative diseases. Mol. Neurobiol. 53, 4094–4125. 10.1007/s12035-015-9337-5 PubMed Abstract | 10.1007/s12035-015-9337-5 | Google Scholar 26198567 PMC4937091

[B43] Park J. S. Ryu J. H. Choi T. I. Bae Y. K. Lee S. Kang H. J. (2016). Innate color preference of zebrafish and its use in behavioral analyses. Mol. Cells 39 (10), 750–755. 10.14348/molcells.2016.0173 PubMed Abstract | 10.14348/molcells.2016.0173 | Google Scholar 27802373 PMC5104883

[B44] Parng C. Seng W. L. Semino C. McGrath P. (2002). Zebrafish: A preclinical model for drug screening. Assay. Drug Dev. Technol. 1 (1), 41–48. 10.1089/154065802761001293 PubMed Abstract | 10.1089/154065802761001293 | Google Scholar 15090155

[B45] Peter J. L. Michael D. Arne O. OhmAn A. (2000). Fear and anxiety: Animal models and human cognitive psychophysiology. J. Affect. Disord. 61 (3), 137–159. 10.1016/s0165-0327(00)00343-8 PubMed Abstract | 10.1016/s0165-0327(00)00343-8 | Google Scholar 11163418

[B46] Piato Â. L. Capiotti K. M. Tamborski A. R. Oses J. P. Barcellos L. J. G. Bogo M. R. (2011). Unpredictable chronic stress model in zebrafish (*Danio rerio*): Behavioral and physiological responses. Prog. Neuropsychopharmacol. Biol. Psychiatry 35 (2), 561–567. 10.1016/j.pnpbp.2010.12.018 PubMed Abstract | 10.1016/j.pnpbp.2010.12.018 | Google Scholar 21187119

[B47] Radulovic Jelena Lynn Y. (2019). N-Methyl D-aspartate receptor subunit signaling in fear extinction. Psychopharmacology 236 (1), 239–250. 10.1007/s00213-018-5022-5 PubMed Abstract | 10.1007/s00213-018-5022-5 | Google Scholar 30238131 PMC6374191

[B48] Rajabi S. Ramazani A. Hamidi M. Naji T. (2015). *Artemia salina* as a model organism in toxicity assessment of nanoparticles. Daru 23 (1), 20. 10.1186/s40199-015-0105-x PubMed Abstract | 10.1186/s40199-015-0105-x | Google Scholar 25888940 PMC4344789

[B50] Shah B. Modi P. Sagar S. R. (2020). *In silico* studies on therapeutic agents for COVID-19: Drug repurposing approach. Life Sci. 252 (117652), 117652. 10.1016/j.lfs.2020.117652 PubMed Abstract | 10.1016/j.lfs.2020.117652 | Google Scholar 32278693 PMC7194845

[B51] Sinha S. K. Shakya A. Prasad S. K. Singh S. Gurav N. S. Prasad R. S. (2020). An *in-silico* evaluation of different Saikosaponins for their potency against SARS-CoV-2 using NSP15 and fusion spike glycoprotein as targets. J. Biomol. Struct. Dyn., 1–12. 10.1080/07391102.2020.1762741 PubMed Abstract | 10.1080/07391102.2020.1762741 | Google Scholar PMC7232888 32345124

[B52] Steimer T. (2011). Animal models of anxiety disorders in rats and mice: Some conceptual issues. Dialogues Clin. Neurosci. 13 (4), 495–506. 10.31887/dcns.2011.13.4/tsteimer PubMed Abstract | 10.31887/dcns.2011.13.4/tsteimer | Google Scholar 22275854 PMC3263396

[B53] Stewart A. Wu N. Cachat J. Hart P. Gaikwad S. Wong K. (2011). Pharmacological modulation of anxiety-like phenotypes in adult zebrafish behavioral models. Prog. Neuropsychopharmacol. Biol. Psychiatry 35 (6), 1421–1431. 10.1016/j.pnpbp.2010.11.035 PubMed Abstract | 10.1016/j.pnpbp.2010.11.035 | Google Scholar 21122812

[B54] Stewart A. M. Braubach O. Spitsbergen J. Gerlai R. Kalueff A. V. (2014). Zebrafish models for translational neuroscience research: From tank to bedside. Trends Neurosci. 37 (5), 264–278. 10.1016/j.tins.2014.02.011 PubMed Abstract | 10.1016/j.tins.2014.02.011 | Google Scholar 24726051 PMC4039217

[B55] Stewart A. M. Braubach O. Spitsbergen J. Gerlai R. Kalueff A. V. (2014). Zebrafish models for translational neuroscience research: From tank to bedside. Trends Neurosci. 37 (5), 264–278. 10.1016/j.tins.2014.02.011 PubMed Abstract | 10.1016/j.tins.2014.02.011 | Google Scholar 24726051 PMC4039217

[B56] Ulrich-Lai Y. M. Christiansen A. M. Wang X. Song S. Herman J. P. (2016). Statistical modeling implicates neuroanatomical circuit mediating stress relief by ‘comfort’ food. Brain Struct. Funct. 221, 3141–3156. 10.1007/s00429-015-1092-x PubMed Abstract | 10.1007/s00429-015-1092-x | Google Scholar 26246177 PMC4744589

[B57] Wong K. Elegante M. Bartels B. Elkhayat S. Tien D. Roy S. (2010). Analyzing habituation responses to novelty in zebrafish (*Danio rerio*). Behav. Brain Res. 208 (2), 450–457. 10.1016/j.bbr.2009.12.023 PubMed Abstract | 10.1016/j.bbr.2009.12.023 | Google Scholar 20035794

[B58] Xu Z. Wang F. Tsang S. Y. Ho K. H. Zheng H. Yuen C. T. (2006). Anxiolytic-like effect of baicalin and its additivity with other anxiolytics. Planta Med. 72 (02), 189–192. 10.1055/s-2005-873193 PubMed Abstract | 10.1055/s-2005-873193 | Google Scholar 16491459

[B59] Zhang J. Deng Y. Cheng B. Huang Y. Meng Y. Zhong K. (2020). Protective effects and molecular mechanisms of baicalein on thioacetamide-induced toxicity in zebrafish larvae. Chemosphere 256, 127038. 10.1016/j.chemosphere.2020.127038 PubMed Abstract | 10.1016/j.chemosphere.2020.127038 | Google Scholar 32470728

[B60] Zhou R. Han X. Wang J. Sun J. (2015). Baicalin may have a therapeutic effect in attention deficit hyperactivity disorder. Med. Hypotheses 85 (6), 761–764. 10.1016/j.mehy.2015.10.012 PubMed Abstract | 10.1016/j.mehy.2015.10.012 | Google Scholar 26604025

